# Development and Quality Characteristics of *Jangjorim* Prepared Using Long-Arm Octopus (*Octopus minor*) as an Elderly-Friendly Food

**DOI:** 10.3390/foods12244375

**Published:** 2023-12-05

**Authors:** Sang-In Kang, Jin-Soo Kim, Sun-Young Park, Si-Hyeong Park, Ji-Hoon Park, Mi-Soon Jang, Jae-Young Oh, Jae-Suk Choi

**Affiliations:** 1 Seafood Research Center, Industry-Academic Cooperation Foundation, Silla University, 606, Advanced Seafood Processing Complex, Wontang-ro, Amanam-dong, Seo-gu, Busan 49277, Republic of Korea; ftrnd5@silla.ac.kr; 2 Department of Seafood Science and Technology, Institute of Marine Industry, Gyeongsang National University, 2-9, Tongyeonghaean-ro, Tonyeong-si 53064, Republic of Korea; jinsukim@gnu.ac.kr (J.-S.K.); tjsdud3591@gnu.ac.kr (S.-Y.P.); sipark@gnu.ac.kr (S.-H.P.); rnd-jh@gnu.ac.kr (J.-H.P.); 3 Food Safety and Processing Research Division, National Institute of Fisheries Science, 216, Gijanghaean-ro, Gijang-eup, Busan 46083, Republic of Korea; suni1@korea.kr (M.-S.J.); ojy0724@korea.kr (J.-Y.O.)

**Keywords:** long-arm octopus, *Jangjorim*, elderly-friendly food, texture-modified technology, sensory texture properties, home meal replacement (HMR)

## Abstract

We prepared a long-arm octopus *Jangjorim* prototype (LOJP) by optimizing the ratio of ingredients for seasoning and establishing heat sterilization parameters. The optimal amounts of purified water (2.9–56.6%, A), starch syrup (0.3–37.8%, B), and soy sauce (25.5–71.5%, C) for the production of seasoning soy sauce were obtained using response surface analysis. The LOJP was prepared by combining A, B, and C under the optimal conditions and evaluated for consumer preferences and physicochemical, nutritional, and microbiological properties and compared with Korea’s legal management standards for geriatric nutrition. The hardness of the LOJP produced using the optimal mixing ratio of purified water (51.2%, 154.0 g), starch syrup (29.3%, 308.0 g), and soy sauce (19.5%, 256.9 g) was 36.7 × 1000 N/m^2^. This value was lower than the hardness of raw octopus (2153.6 × 1000 N/m^2^) by 2116.9 × 1000 N/m^2^. It received the highest score (8.7) in the preference evaluation of older consumers. The LOJP was classified as level 2, allowing consumption through the gums of elderly consumers per Korea’s food standards for the elderly. The LOJP was the product highly preferred by elderly consumers with chewing disorders due to its ease of intake and nutritional content.

## 1. Introduction

The National Health and Nutrition Examination (2005) [1] reported that Koreans consume an average of 1291.4 g of food per person per day. This consumption includes 76.2 g (5.9%) of seafood products, which is gradually increasing every year.

According to the Korea Rural Economic Institute, the per capita consumption of seafood in Korea increased from 52.8 kg in 2001 to 68.4 kg in 2020. This increase in the consumption of aquatic products is likely attributable to the improvement in national income and the general interest among the population in consuming healthy food. This has increased consumer preference for aquatic products as healthy foods.

Cephalopods are among the most important aquatic resources worldwide due to their excellent taste and high nutritional value. Korea consumes more than 400,000 tons of cephalopods annually. The Korea Fishery Trade Association reports that cephalopod imports in Korea have been steadily increasing since 2017 [2,3].

Based on the datasets provided by the National Statistical Office of the Republic of Korea, the proportion of the elderly population was recorded at 7.0% in the year 2000, signifying the onset of a demographic transition towards an aging society. This reached 14.2% in 2017, which indicated a transition into an aged society. By 2025, it is predicted that the elderly population will reach 20.6%; thus, the country will enter into being a super-aged society [4].

Recently, due to the increase in the number of elderly citizens in Korea, Korean food factories are also producing elderly-friendly foods. Standards for elderly-friendly foods are presented and managed by the Food Code [5] of the Ministry of Food and Drug Safety and the Korean Industrial Standards (KS standards) [6] of the Ministry of Trade, Industry and Energy. Specifically, the Ministry of Agriculture, Food, and Rural Affairs and the Ministry of Oceans and Fisheries have recently designated excellent elderly-friendly foods; most of the 79 types of these excellent elderly-friendly foods are mainly agricultural or livestock products, and only eight products are related to aquatic products. These aquatic products use flatfish, hairtail, cod, abalone, seaweed, pollack, mackerel, and bonito flakes. However, no elderly-friendly food using octopuses (small octopus or long-arm octopus), preferred by Koreans, has been released.

In Korea, long-arm octopuses are mainly consumed in stir-fried, steamed, and *yeonpo-tang* (octopus soup) form, and processed foods such as stir-fried and canned foods have been studied [7,8]. However, to the best of our knowledge, there is no elder-friendly food that uses a long-arm octopus.

*Jangjorim* is a Korean food prepared by boiling meat or eggs in soy sauce [9]. Starting with the 1988 Seoul Olympics, the number of people in the West who became aware of *Jangjorim* has increased, and it is known as the most popular Korean dish among foreigners, along with *Galbitang* (beef ribs Korean soup), *Samgyetang* (chicken and ginseng Korean soup), and *Bibimbap* (Korean food composed of steamed rice, minced beef, and other vegetables along with *Gochujang* (a red chili paste) and sesame seed oil). In East Asia, many people like *Jangjorim* because there are similar dishes in Chinese and Japanese cuisines, and their tastes are quite similar. Raw materials for making soybean paste for *Jangjorim* include beef [10,11], pork [12], and chicken meat [13] for meat dishes, and chicken eggs [14] and quail eggs [15] for egg dishes. However, to the best of our knowledge, *Jangjorim* has rarely been developed using seafood as raw meat.

Long-arm octopus is known to be a representative seafood product that helps strengthen immunity due to its high taurine content so it is receiving great attention from elderly consumers. However, it is difficult for elderly consumers with chewing disorders to consume it raw or after simple heat treatment.

Therefore, in this study, we identified a method of softening the physical properties of long-arm octopus, which has hard muscle tissue, to make it easier for elderly consumers to consume and investigated the quality characteristics of Jangjorim developed using long-arm octopus.

## 2. Materials and Methods

### 2.1. Materials

Frozen long-arm octopus (*Octopus minor*; 500 g/block, average octopus weight 5.0 g ± 0.8 g) imported from China in May 2022 was purchased from Wonil Seafood Co., Ltd. (Busan, Republic of Korea) and used for prototype production and subsequent experiments. The frozen long-arm octopus arms were thawed under running water (30 min) before use. The ingredients used to prepare the prototype of the long-arm octopus *Jangjorim* were konjac (Sajo Daerim Co., Ltd., Seoul, Republic of Korea), starch syrup (CJ Cheil Jedang Co., Ltd., Seoul, Republic of Korea), cooking alcohol (rice wine for cooking; Lotte Chilsung Beverage Co., Ltd., Seoul, Republic of Korea), sugar (CJ Jeil Jedang Co., Ltd., Seoul, Republic of Korea), soy sauce (Daesang Co., Ltd., Seoul, Republic of Korea), and *Cheongyang* hot pepper (purchased from a large market in Tongye, Gyeongsangnam-do, Republic of Korea).

### 2.2. Optimization of the Composition Ratio of Soy Sauce Mixture Using Response Surface Analysis (RSM)

To optimize the composition ratio of the soy sauce mixture, purified water (A), starch syrup (B), and soy sauce (C) were considered independent variables, and a prediction model was designed using MINTAB software (MINITAB 18, MINITAB LLC., Pine Hall Rd State College, PA, USA) after encrypting the central point of the mixing ratio using the following conversion formula: [*X*_1_: A/(B + C), *X*_2_: B/C] (Table 1). According to the Central Composite Rotatable Design (CCRD) [16], *X*_1_ and *X*_2_ were coded into five levels based on the minimum and maximum values of the preliminary experimental results. Then, 11 samples of the long-arm octopus *Jangjorim*, to which various types of soy sauce mixtures were added, were prepared, and the experiment was conducted (Table 2). The dependent variables for the mixing ratio conditions of the long-arm octopus *Jangjorim* were hardness (Y_1_), salinity (Y_2_), and sensory taste score (Y_3_), which were used for the regression analysis.

$$Y=\beta_{0}\sum_{i=1}^{2}\beta_{i}X_{i}+\sum_{i=1}^{2}\beta_{ij}{X_{i}}^{2}+\sum_{i=1}^{1}\sum_{j=i+1}^{2}\beta_{ij}X_{i}X_{j}$$

where *Y* is the dependent variable; *β*_0_ is a constant; *β*_i_, *β*_ii_, and *β*_ij_ are regression coefficients; and *X*_i_ and *X*_j_ are independent variables.

### 2.3. Manufacture of a Long-Arm Octopus Jangjorim Prototype (LOJP)

**Figure 1:**
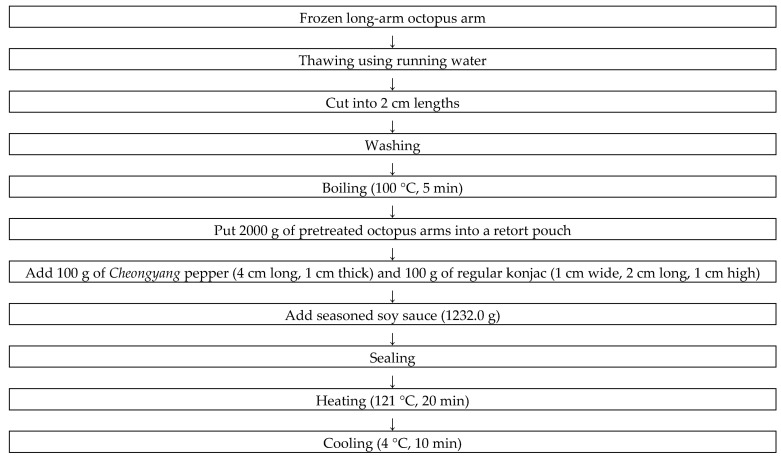
Manufacturing process diagram of long-arm octopus prototype.

The frozen long-arm octopus was thawed under running water for 30 min, and the arms were cut at 2 cm intervals, washed again in running water for 1 min, and boiled at 100 °C for 5 min. Then, the boiled long-arm octopus arm (2000 g) was placed into a retort pouch (nylon (inner layer)–aluminum (middle laye])–polyethylene terephthalate (exterior); 20 cm × 27 cm; DnP Co., Ltd., Gwangju, Gyeonggi-do, Korea). To prepare the soy sauce mixing solution, purified water (154 g (2.9–56)6%)), starch syrup (308.0 g (0.3–37)8%)), soy sauce (256.9 g (25.5–71)5%)), cooking alcohol (154 g), and starch water (359.1 g) were put in a boiling pot (diameter 26.8 cm × height 13.5 cm) and heated (100 °C, 5 min). *Cheongyang* pepper was cut to 4 cm long and 1 cm wide, konjac was cut (1 cm wide × 2 cm long × 1 cm height), and 10.0 g of coconut oil and 100 g of *Cheongyang* pepper and konjac were placed in a retort pouch. The retort pouch (total content 3432 g/3 pack) was filled and sealed, and then the pouch was heated (121 °C, 20 min) in retort sterilization equipment (DWRETO-ACE-200 L; Hyosung FMT Corp., Daegu, Korea) and then cooled to produce a prototype (Table 3, Figure 1).

### 2.4. Hardness

The hardness analysis was conducted using a texture meter (CT3-1000, Brookfield, Middleboro, MA, USA) according to the following methods: the “Experiment method 1” as per the the Food Code [5] and KS standards [6] and “Experiment method 3” as per the KS standards (Figure S1).

### 2.5. Salinity

Analysis of salinity was conducted based on the method of the Food Code [5] and confirmed by referring to the method of Kang et al. [17].

### 2.6. Volatile Basic Nitrogen (VBN)

VBN content was measured by the method described in the Food Code [5] and the method of Kang et al. [17].

### 2.7. Moisture, Crude Protein, Ash, Crude Lipid, Carbohydrates, and Calories

For the quantification of proximate components, around 0.5 g of the finely ground sample underwent analysis. The moisture content was determined using the atmospheric pressure drying technique, crude protein was quantified through the semimicro Kjeldahl procedure, crude lipid content was ascertained via the Soxhlet extraction method, and ash content was measured using the dry ashing technique, all in accordance with the specifications outlined in the Food Code [5]. Carbohydrate content was calculated as a percentage of the content excluding the sum of the moisture, ash, crude protein, and crude lipid content. Calorie content was derived by referring to the energy conversion coefficient of the Japanese food ingredient table and standard ingredient analysis data [18].

### 2.8. Total Amino Acids

Total amino acid content was analyzed by referring to the method described in the Food Code [5] and the method of Kang et al. [17].

### 2.9. Fatty Acids

The analysis preparation of the fatty acid profiles was conducted by referring to the method of Bligh and Dyer [19], and the identification of the fatty acid profile was conducted in alignment with the methodology delineated by Moon et al. [20].

### 2.10. Mineral

Mineral content of the samples was quantified based on the protocol stipulated in the Food Code [5]. For this assessment, a 1 g aliquot of the sample underwent pretreatment via the wet digestion technique, subjected to elevated temperature and reduced pressure conditions. Subsequent inorganic determinations were executed using inductively coupled plasma mass spectrometry (X Series II, Thermo Fisher Scientific, Waltham, MA, USA).

### 2.11. Total Bacterial Counts (TBCs)

TBCs were determined according to the AOAC method [21]. Diluted homogenates from the samples were spread onto dehydrated experimental film (3M petrifilm aerobic count plates, Maplewood, MN, USA) and subsequently incubated at a temperature of 37 ± 1 °C. Colonies manifesting either a reddish hue or gas production were enumerated as TBC-positive entities.

### 2.12. Bacterial Growth Counts (BGC)

Bacterial growth tests were performed according to the methods described in the Food Code [5]. Samples to determine bacterial growth included five LOJPs treated at 121 °C for 15, 20, 25, and 30 min, respectively. The five samples were cultured in unopened containers and packaging in an incubator (J-NB2, JISICO Co., Seoul, Korea) for 10 days (36 ± 1 °C) and then incubated at room temperature (25 ± 1 °C) for 1 d. It was left unattended. At this time, it was observed that the container had expanded, and it was judged as positive or negative; if it was negative, the following bacterial test was performed. To prepare the pretreatment samples, the surfaces of the five samples packaged and sealed in retort pouches were wiped with 70% alcohol and opened, and 25 g of the contents was added to 225 mL of the diluent and homogenized. One milliliter of the homogenate was collected in a sterile test tube, diluted, mixed with 9 mL of the diluent, and used as the test solution. The diluted solution was inoculated into 5 thioglycollate media (Floid Thioglycollate Medium, MICROGIENE Co., Ltd., Gunpo, Gyeonggi-do, Republic of Korea) at 1 mL each and incubated at 36 ± 1 °C for 48 h, and if bacterial growth was confirmed in any one of the five samples, the test was judged to be positive.

### 2.13. Heavy Metals

Pretreatment and analysis of samples for Pb, Cd, total As, and total Hg analysis were conducted in ICP-MS (NexION300, PerkinElmer, Waltham, MA, USA) according to the sequence described in the Food Code [5]. Both standard materials and CRM (DORM-4, National Research Council, Ottawa, On, Canada) underwent identical analytical processes.

### 2.14. Sensory Evaluation

The sensory assessment of the formulated LOJP was undertaken within the elderly demographic, post-securing research clearance for human subjects under the identifier GIRB-G22-Y-0058. This approval was granted by the Institutional Review Board of GNU, Jinju, South Korea, in congruence with the stipulations of the Enforcement Decree of the Bioethics and Safety Act. The sensory evaluation employed a hedonic scale and incorporated the feedback of 28 respondents (comprising an equal split of 14 females and 14 males, all aged between 65 and 75 years with a median age of 70 years) domiciled at the Senior Care Center, Tongyeong, South Korea. Assessment metrics encompassed visual appeal, gustatory profile, aromatic qualities, tactile attributes, and holistic acceptance, adhering to methodologies proposed by Lawless and Heymann [22] and further expanded by Kang et al. [17].

### 2.15. Structural Analysis by Scanning Electron Microscopy

The ultrastructural morphology of the long-arm octopus muscle tissue was elucidated utilizing a scanning electron microscope, adhering to protocols delineated by Rattanasatheirn et al. [23] and further expanded by Kang et al. [17].

### 2.16. Statistical Analysis

To calculate the standard deviation and significant difference of the result data, the analysis of variance (ANOVA) was conducted using SPSS statistical software (SPSS 10.1), and Duncan’s multiple test was performed after ANOVA.

## 3. Results

### 3.1. Statistical Correlation between Material Mixing Ratio and Sensory Elements

**Figure 2:**
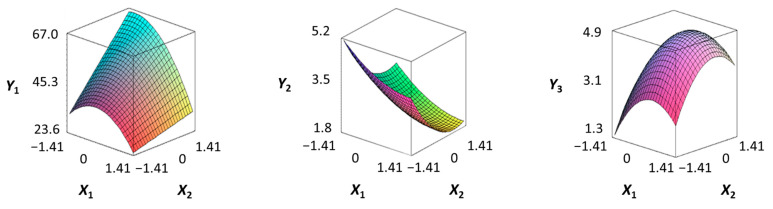
Three-dimensional response surface plots for preparing restructured long-arm octopus *Jangjorim* based on the hardness (*Y*_1_), salinity (*Y*_2_), and sensory taste (*Y*_3_).

To examine the relationship between the independent (*X*_1_ and *X*_2_) and dependent variables (*Y*_1_, *Y*_2_, and *Y*_3_), RSREG was performed using the MINITAB statistical program. The relationship between the two independent variables and the dependent variable is shown in Figure 2 as a three-dimensional graph created using Maple software (MAPLE Ver. 12). The hardness (*Y*_1_) of the long-arm octopus *Jangjorim* showed a tendency to gradually decrease as the code value for *X*_1_ moved from −1.414 to 0.09 and then gradually increased, and, in the case of *X*_2_, it showed a tendency to increase as it moved from −1.414 to +1.414. The salinity (*Y*_2_) of the long-arm octopus *Jangjorim* tended to decrease as it moved from −1.414 to +1.414 in the case of *X*_1_, and, in the case of *X*_2_, it tended to decrease as it moved from −1.414 to +0.61 and then gradually increased. The sensory taste score (*Y*_3_) of the long-arm octopus *Jangjorim* increased rapidly from −1.414 to 0.30 in *X*_1_ and from −1.414 to 0.44 in *X*_2_, reaching a maximum, after which it showed a sharp decreasing trend (Figure 2).

### 3.2. Sensory Properties of Long-Arm Octopus Jangjorim by Ingredients (Water, Starch Syrup, Soy Sauce)

According to the mixing ratio of long-arm octopus *Jangjorim* (purified water (A), starch syrup (B), and soy sauce (C)), the coefficients for the quadratic equation related to the linear term, quadratic term, and cross term of the dependent variables (hardness (*Y*_1_), salinity (*Y*_2_), and sensory taste score (*Y*_3_)) and their significance are shown in Table 4. In the case of longitude (*Y*_1_), only the following four terms were recognized as significant: linear terms *X*_1_ and *X*_2_, quadratic terms *X*_1_ *X*_1_, and cross term *X*_1_*X*_2_ (*p* < 0.05). However, in the case of the remaining quadratic term (*X*_2_*X*_2_), significance was not observed (*p* > 0.05). For both salinity (*Y*_2_) and sensory taste scores (*Y*_3_), only four terms, namely, the linear terms *X*_1_ and *X*_2_ and the quadratic terms *X*_1_*X*_2_ and *X*_2_*X*_2_, were recognized as significant (*p* < 0.05); however, in the case of the remaining terms, the cross term (*X*_1_*X*_2_) was not significant (*p* > 0.05). The significance (*p* < 0.05) of the response model equation for hardness (*Y*_1_), salinity (*Y*_2_), and sensory taste score (*Y*_3_) is expressed in the equation as follows: *Y*_1_ = 37,4—0 − 6526*X*_1_ + 6659*X*_2_ + 4578*X*_1_*X*_1_ − 2984*X*_1_*X*_2_ (R^2^ = 0.955, *p*-value = 0.000), *Y*_2_ = 2.53—3 − 0.4725*X*_1_ − 0.6755*X*_2_ + 0.1646*X*_1_*X*_1_ + 0.3896*X*_2_*X*_2_ (R^2^ = 0.973, *p*-value = 0.000), and *Y*_3_ = 4.600 + 0.660*X*_1_ + 0.945*X*_2_ − 1.013*X*_1_*X*_2_ − 1.063*X*_2_*X*_2_ (R^2^ = 0.927, *p*-value = 0.000) [24].

Table 5 shows the correlations between the independent and dependent variables required to optimize the mixing ratio of purified water (A), starch syrup (B), and soy sauce (C) to create the LOJP. When confirming the significance of the reaction model equation for optimizing the mixing conditions of purified water (A), starch syrup (B), and soy sauce (C), in the case of hardness (*Y*_1_), the linear, quadratic, and cross terms were all significant (*p* < 0.05), whereas, in the case of salinity (*Y*_2_) and sensory taste score (*Y*_3_), only the linear and quadratic terms were significant (*p* < 0.05). The *p*-value for the lack-of-fit test indicated the suitability of the response model equation for hardness (*Y*_1_), salinity (*Y*_2_), and sensory taste score (*Y*_3_) for optimizing the mixing conditions of purified water (A), starch syrup (B), and soy sauce (C), with a hardness of 0.152, salinity of 0.114, and sensory taste score of 0.154, all of which were higher than 0.05. In addition, the coefficients of determination (R^2^) were 0.955 for hardness, 0.973 for salinity, and 0.927 for the sensory taste score; all of the R^2^ values were close to 1. The *p*-value of the model was 0.000 for hardness, 0.000 for salinity, and 0.001 for the sensory taste score, all of which were lower than 0.05, indicating that all designed models were suitable [25].

### 3.3. Optimization of Mixing Ratio of Materials for Long-Arm Octopus Jangjorim

The results of predicting the optimal values of all three mixing ratios (independent variables) for the sensory characteristics (dependent variables) of long-arm octopus *Jangjorim* are as follows: the core values of the independent variables (*X*_1_ and *X*_2_) that simultaneously met the dependent variables of long-arm octopus *Jangjorim* were 0.07 and 0.00, and, when converting them into actual values, they were 1.05 and 1.50, respectively (Table 6). When converting these encoded values into actual addition ratios, the mixing ratios of purified water (A), starch syrup (B), and soy sauce (C) were 51.2%, 29.3%, and 19.5%, respectively (Table 6). Furthermore, the hardness of the four long-arm octopus *Jangjorim* prototypes manufactured under these conditions was 37.0 × 1000 N/m^2^, the salinity was 2.5 g/100 g, and the sensory taste score was predicted to be 4.7 points.

The actual measured sensory characteristics, namely, hardness, redness, and overall acceptability, were found to be 27.8 × 1000 N/m^2^, 10.29, and 4.2 points, respectively, for long-arm octopus *Jangjorim* manufactured by applying the optimal mixing ratio (purified water 51.2%, starch syrup 29.3%, soy sauce 19.5%) derived through RSM. These results were not significantly different from the predicted values (hardness, 36.7 × 1000 N/m^2^; salinity 2.5 g/100 g; sensory taste score 4.6 points) (*p* > 0.05) (Table 7) and were confirmed to be the optimal model for optimizing the mixing ratio of purified water, starch syrup, and soy sauce in the soy sauce mixture for long-arm octopus *Jangjorim*.

### 3.4. Thermal Death Time (F_0_) Value and Hardness According to Sterilization Time

**Figure 3:**
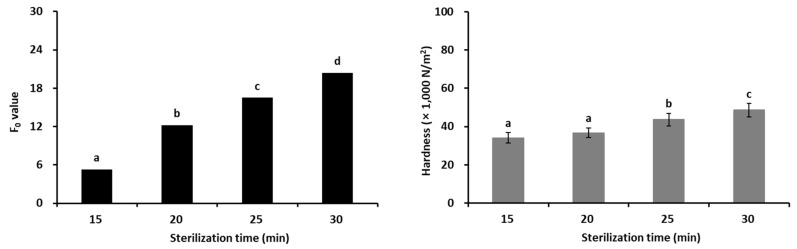
F_0_ value and hardness (×1000 N/m^2^) by sterilization time of long-arm octopus *Jangjorim*. Different letters (a–d) by data on the bar indicate significant differences at *p* < 0.05.

The results of the F0 value and hardness according to the heating time of the LOJP are shown in Figure 3. The LOJP, manufactured with the optimal mixing ratio derived through RSM, was determined to have been sufficiently sterilized even with a sterilization time of 15 min. However, considering the variation in air emissions from the retort, a longer time of 20 min (121 °C) was found to be the most optimal sterilization condition. When the LOJP prepared by adding the optimal soy sauce mixture was treated under different heat treatment conditions (15 min, 20 min, 25 min, and 30 min at 121 °C), the results of examining the hardness characteristics of these prototypes were as shown in Figure 3. The hardness of long-arm octopus *Jangjorim* was 34.1 × 1000 N/m^2^ for 15 min, 36.7 × 1000 N/m^2^ for 20 min, 43.6 × 1000 N/m^2^ for 25 min, and 48.6 × 1000 N/m^2^ for 30 min, showing the tendency to increase as the degree of heat treatment increased, but no significant difference was found between the sterilization times of 15 and 20 min (*p* < 0.05).

### 3.5. Proximate Composition, Calories Contents, Salinity

Analysis of the general ingredients of the raw long-arm octopus and LOJP revealed 80.0% moisture, 9.7% crude protein, 4.2% crude fat, 1.7% ash, and 4.2% carbohydrates (Table 8).

The calorific value of the raw long-arm octopus and LOJP was 96.6 kcal (Table 8), and the value corresponded to 4.8% for men and 6.0% for women, respectively, compared with the estimated energy requirements for those aged 65–74 (2000 kcal for men and 1600 kcal for women) [26].

The salinity was approximately 0.8 g lower than the sufficient salt intake per 1 day for Korean women and men aged 65–74 years (3.30 g for women and men), as specified by Korea’s Ministry of Health and Welfare [26] (Table 8).

### 3.6. Total Amino Acid Content

The total amino acid contents of the raw long-arm octopus and LOJP are shown in Table 9. The total amino acid content of the LOJP was 8824.7 mg, and the major amino acids (more than 8% composition) were lysine (707.3 mg, 8.0%), aspartic acid (966.1 mg, 10.9%), glutamic acid (1417.4 mg, 16.1%), and glycine (864.5 mg, 9.8%). The total essential amino acid content of the LOJP was 4038.4 mg, equivalent to 45.7% of the total amino acid content. The LOJP contained 440.1 mg (5.0%) and 707.3 mg (8.0%) of threonine and lysine, respectively, which are restricted amino acids in cereals.

### 3.7. Mineral Content

The mineral contents of the long-arm octopus and LOJP are listed in Table 10. The calcium content of long-arm octopus was 19.1 mg/100 g, potassium content was 144.2 mg/100 g, iron content was 3.8 mg/100 g, and zinc content was 1.1 mg/100 g. The calcium content of LOJP was 23.7 mg/100 g, potassium content was 375.7 mg/100 g, iron content was 4.6 mg/100 g, and zinc content was 2.4 mg/100 g, which were significantly higher than those of long-arm octopus (*p* < 0.05).

### 3.8. Fatty Acid Content

The fatty acid contents of the raw long-arm octopus and LOJP are shown in Table 11. The total fatty acid content of the LOJP was 2562.4 mg, corresponding to 61.0% of the total lipid content. The fatty acid content and composition of the lipids of the LOJP were highest in saturated fatty acids at 1277.5 mg (49.9%), followed by polyunsaturated fatty acids (1114.0 mg, 43.5%); monounsaturated fatty acids were the lowest at 170.9 mg (6.6%). The major fatty acids constituting the lipids of the LOJP were 16:0 (344.4 mg and 13.4%), 18:0 (383.5 mg, 15.0%), 22:0 (413.1 mg, 16.1%), 20:5n-3 (263.2 mg, 10.3%), and 22:6n-3 (626.4 mg, 24.5%).

### 3.9. Freshness Indicators

The pH, which was an indicator of the freshness of the raw long-arm octopus and LOJP, is shown in Table 12. The pH of the raw octopus was 6.60, and that of LOJP was 6.87, indicating a statistically significant difference (*p* < 0.05).

The VBN content, an indicator of the freshness of the raw long-arm octopus and LOJP, is shown in Table 12. The VBN content of the raw octopus was 11.1 mg/100 g, and the VBN content of the LOJP was 15.4 mg/100 g, indicating a statistically significant difference (*p* < 0.05).

The TBC, which represented the quality change index of the raw long-arm octopus and the LOJP, is shown in Table 12. The TBC of the raw long-arm octopus was 1.3 × 10^3^ CFU/g, indicating an average level of freshness. The general bacterial count of the LOJP was determined to be undetectable because it was sterilized.

Table 12 shows the results of measuring the degree of bacterial growth through sterilization treatment of the raw long-arm octopus and LOJP. The raw-material octopus was not measured, and the bacterial growth of the LOJP was undetectable.

### 3.10. Heavy Metals

Table 12 shows the results of measuring the heavy metal contents of the raw long-arm octopus and LOJP. The Pb and Cd contents of the raw octopus were 0.01 mg/kg and 0.04 mg/kg, respectively, and the Pb and Cd contents of the LOJP were 0.002 mg/kg and 0.003 mg/kg, respectively. Korea’s Food Code [5] suggests that among the heavy metal content standards for octopus, the lead content should be 2.0 mg/kg or less and the cadmium content 3.0 mg/kg or less. Therefore, the long-armed octopus used in this experiment was judged to have safe levels of Pb and Cd.

### 3.11. Microstructure Observation by SEM

**Figure 4:**
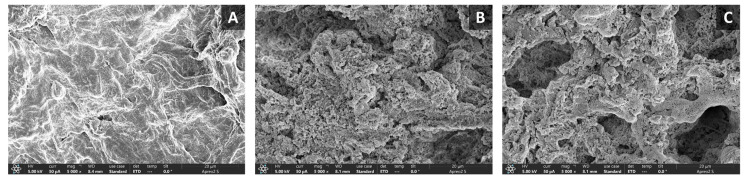
Scanning electron microscope images (magnification: ×5000) of raw long-arm octopus (**A**) and boiled long-arm octopus arm (**B**) and sterilized long-arm octopus arm (**C**) of long-arm octopus *Jangjorim* prototype (LOJP).

An image of the microstructures of the long-arm octopus and the LOJP is shown in Figure 4. The microstructure (A) of the raw long-arm octopus muscle was composed of dense muscle fiber tissue, and the microstructure (B) of the LOJP manufactured only through boiling treatment showed partial cracks between the muscle fibers. The microstructure (C) of the LOJP after retort heating (121 °C, 20 min) showed that a gap of approximately 20 μm had opened between the muscle fiber tissues. Thus, it was concluded that the physical properties of the LOJP were softened by significantly widening the gap between muscle fiber tissues.

### 3.12. Sensory Evaluation of the Prototypes

Table 13 shows the results of a preference evaluation performed among elderly consumers for raw long-arm octopus (sample A), boiled long-arm octopus *Jangjorim* (sample B), and retort-heated raw LOJP (sample C). Preference for appearance was highest for sample C at 8.4 points, which was not significantly different from that for sample B (*p* > 0.05), and sample B showed no significant difference from sample A (*p* > 0.05). Furthermore, sample C had the highest score of 8.1 points for taste, but no significant difference was found compared with that of sample B (*p* > 0.05). Although sample C had the highest preference for flavor at 8.0 points, it did not show a significant difference from sample B (*p* > 0.05); however, sample B showed a significantly higher preference than sample A at 7.6 points (*p* < 0.05). In their preference evaluation, sample C exhibited the highest preference for texture, which is the most important item for older consumers, with 8.7 points, sample B had the second highest preference (7.3), and sample A had the lowest preference (4.2 points). Texture preferences showed significant differences (*p* < 0.05). It was concluded that processed seafood with a soft texture can be provided to elderly consumers by retort-heating raw long-arm octopus to produce a LOJP.

## 4. Discussion

Recent studies on marine-product-based home meal replacement (HMR) products in Korea have introduced HMR foods or elderly-friendly foods such as salmon, chub mackerel, hairtail, pollack, flatfish, Pacific anchovy, and abalone [27,28,29,30,31,32]. Among these, Sutikno et al. [33] studied the development of HMR products using a mollusk, namely, web-foot octopus (*Amphioctopus* sp.), Negara et al. [34] focused on improving quality characteristics of HMR products using common squid (*Todarodes pacificus* Steenstrup), Kang et al. [17] studied texture-modified octopus (*Octopus vulgaris*) arms, and Kang et al. [35] conducted a study on the development of an elderly-friendly HMR product using common squid (*Todarodes pacificus*). However, to date, no studies have reported the development of a *Jangjorim* prototype using the long-arm octopus (*Octopus minor*); therefore, it was developed in this study.

*p*-values for the lack of fit of the design model’s equation for hardness (*Y*_1_), salinity (*Y*_2_), and sensory taste (*Y*_3_) according to the mixing ratio of the materials of the LOJP were 0.152, 0.114, and 0.154, respectively. These values were all > 0.05, and the coefficients of determination (R^2^) for hardness (*Y*_1_), salinity (*Y*_2_), and sensory taste (*Y*_3_) were 0.955, 0.973, and 0.927, respectively, which were all close to 1. The *p*-values of the response surface models for hardness (*Y*_1_), salinity (*Y*_2_), and sensory taste (*Y*_3_) were all 0.000, which were all < 0.05 and confirmed the suitability of the three designed models. These results are consistent with those of the research designed using RSM by Zhou and Regenstein [36] and Bezerra et al. [37].

Thus, the change in taste according to the ratio of the main ingredients of the LOJP determined through RSM showed an inverse tendency in salinity and sensory taste because of the influence of starch syrup and soy sauce. These findings indicate that, as their addition ratio increased, the preference score decreased. These results are consistent with those of Heo and Lee [38] and Kremer et al. [39], who also reported that the addition of ingredients with high salt content, such as soy sauce, reduces preference. Therefore, the results of this study provide evidence that the optimal mixing ratio should be based on consumer preferences.

The optimal sterilization time that can Ie inferred from the F_0_ value of the LOJP was determined to be 20 min. Although the hardness results of the LOJP showed no significant difference between the LOJPs sterilized for 15 and 20 min, when consumed by elderly consumers, 20 min was judged to be appropriate considering safety. Kim et al. [40] conducted a study to establish sterilization conditions for canned goods using skipjack tuna (*Katsuwonus pelamis*) and reported that sterilization must be performed, indicating that the F_0_ value should be ≥4 min to ensure safety during distribution and storage. These findings are consistent with the results presented in this study.

Presumably, the longer the heating treatment time of the LOJP, the higher the hardness of the LOJP because the sauce and long-arm octopus heated for a long time under high-temperature conditions tended to clump together. Furthermore, it was determined that the pectin, sugars, and organic acids in starch syrup and soy sauce were gelled by heat treatment. These results are similar to those reported by Choi et al. [41], who studied changes in quality characteristics during refrigerated storage of soy sauce manufactured using superheated steam and ultra-high-pressure processing. Accordingly, in this study, the sterilization time (20 min) was established at a level where the hardness of the LOJP did not interfere with the masticatory function of elderly consumers.

The comparison of the crude protein of the LOJP and the total amino acid content showed that the crude protein content of the LOJP (8.7 g/100 g) increased by 11.5% compared with that of the raw long-arm octopus (9.7 g/100 g) (*p* < 0.05). Additionally, the total amino acid content of the LOJP was 8824.7 mg/100 g, which was 6.3% higher than that of the raw long-arm octopus (8299.9 mg/100 g); however, the difference was not significant. The difference between these results was due to the addition of soy sauce as an ingredient in the LOJP. Soy sauce is generally manufactured through the fermentation of soy protein and is a seasoning with <10% protein content [42]. Wang et al. [43] reported that the protein contained in soy sauce powder can be used as an ingredient and a nutritional source for various foods.

The fatty acid content in the raw long-arm octopus and the LOJP was confirmed to be made up of 22 (11:0–22:6n-3) and 24 (4:0–22:6n-3) fatty acids, respectively. The main fatty acids in the raw long-arm octopus were 22:6n-3 (EPA, 78.7 mg/100 g), 20:5n-3 (DHA, 79.8 mg/100 g), 16:0 (palmitic acid, 56.5 mg/100 g), and 18:0 (stearic acid, 23.2 mg/100 g). Among the total fatty acid contents of raw long-arm octopus, the percentage of polyunsaturated fatty acid was the highest at 163.7 mg/100 g (48.6%), followed by saturated fatty acid (SFA) at 137.3 mg/100 g (40.8%).

The main fatty acids of the LOJP were C22:6n-3 (EPA, 626.4 mg/100 g), C23:0 (413.1 mg/100 g), C18:0 (stearic acid, 383.5 mg/100 g), C16:0 (palmitic acid, 344.4 mg/100 g), and C20:5n-3 (DHA, 263.2 mg/100 g), which were different when compared to the fatty acid composition of raw long-arm octopus. Regarding the overall fatty acid content of the LOJP, the proportion of SFA was the highest at 1277.5 mg/100 g (49.9%), followed by PUFA at 1114.0 mg/100 g (43.5%), and monounsaturated fatty acid was 170.9 mg/100 g (6.6%), which accounted for the lowest proportion.

The ω-3 fatty acid content in LOJP was 914.0 mg/100 g (35.8%). This was confirmed to be higher by 751 mg/100 g compared to the content of ω-6 fatty acid (163.0 mg/100 g, 6.3%). Panpipat and Yongsawatdigul [44] and Kang et al. [17] reported that nutritionally balanced foods contain relatively higher levels of ω-3 fatty acids than ω-6 fatty acids. Foods typically contain various fatty acids, but studies support that a higher ω-6 fatty acid content can cause disease [44,45]. The results of the fatty acid content of the LOJP suggest it to be an excellent food that can provide nutrients to elderly consumers through ω-3 fatty acids.

The mineral content of long-arm octopus was compared with the nutritional intake standards for elderly age Korean consumers (65–74 years old), as reported by Korea’s Ministry of Health and Welfare [26]. It was 2.7% for men and 2.4% for women compared with the recommended daily calcium intake (700 mg/day for men and 800 mg/day for women). It was 4.1% of the standard daily potassium intake (3500 mg/day for both men and women) for both men and women. Compared with the recommended daily iron intake (9 mg/day for men and 8 mg/day for women), it was 42.2% for men and 47.5% for women, and it was 12.2% for men and 15.7% for women when compared with the recommended daily zinc intake (9 mg/day for men and 7 mg/day for women).

In contrast, the calcium content of the LOJP was 23.7 mg/100 g, potassium content was 375.7 mg/100 g, iron content was 4.6 mg/100 g, and zinc content was 2.4 mg/100 g. This was 3.4% for men and 3.0% for women compared with the recommended daily calcium intake according to the nutritional intake standards for elderly age Korean consumers (65–74 years old). This was 10.7% for both men and women compared with the standard daily potassium intake. The recommended daily iron intake was 51.1% for men and 57.5% for women. Compared with the recommended daily zinc intake (9 mg/day for men and 7 mg/day for women), this was 26.7% for men and 34.3% for women. Therefore, the mineral content of the LOJP was higher than that of the raw long-arm octopus, and the LOJP was determined to be a major source of minerals.

The LOJP had a pH of 6.87 and a VBN content of 15.4 mg/100 g, indicating excellent freshness. Additionally, when the LOJP was produced using raw long-arm octopuses, TBC and BGA were not detected in the LOJP, confirming its safety against bacteria. In particular, it met all the standards presented in Korea’s Food Code [5], indicating that it has hygiene indicators for supplying safe food to elderly consumers. Ramírez-Suárez et al. [46] reported the indicators of most heat-vulnerable bacteria that are present in food. Thus, the heating conditions (121 °C, 20 min) applied to the LOJP developed in this study were confirmed to be suitable conditions for sterilizing the LOJP. Ates et al. [47] reported that no bacteria were detected when fish soup was sterilized for 5.5 min, 6.8 min, and 11.5 min in a retort at 62 °C, 65 °C, and 68 °C.

Additionally, the heavy metal content of the LOJP had low concentrations of Pb (0.002 mg/kg) and Cd (0.003 mg/kg) compared to the standards provided by Korea’s Food Code [5], and it was determined that this amount did not affect the safety of the consumers. Therefore, the microbiological characteristics of the LOJP and the trace heavy metal content results indicate that it is a safe food for elderly consumers.

Yun et al. [31] reported that among marine products, the microstructures of animal raw materials with muscles have different textures depending on the various muscle materials. Hence, the microstructure of animal seafood products can serve as a basis for consumers to consider texture factors when chewing [48,49].

The raw long-arm octopus (sample A) exhibited a very dense tissue structure. This may be possible because the long-arm octopus was not subjected to any heat treatment, and the space between the muscle fibers of the myofibrillar protein was small, resulting in a dense structure. In contrast, the cross-section of the LOJP (sample B) produced using the long-arm octopus that had only been boiled presented with more holes compared with sample A, which were created due to cracking between muscle fibers. The cross-section of the LOJP (sample C) produced using a sterilized long-arm octopus presented more widely spaced muscle fibers. It was determined that the muscle fibers of the long-arm octopus expanded because of the heat treatment, and the muscle tissue became softer. These results were inferred from the texture results reported by Torres-Arreola et al. [50]. Reportedly, after boiling jumbo squid (*Dosidicus gigas*) muscle (hardness: 29.5 N) at 100 °C (for 30 min), the hardness was considerably reduced to 9.1 N, and the muscle became softer. In contrast, in this study, it was confirmed that when raw long-arm octopus (hardness: 2153.6 × 1000 N/m^2^) was heated at 121 °C for 20 min, the hardness was considerably reduced to 34.4 × 1000 N/m^2^. Therefore, it is difficult to find studies on the heat-treatment-induced softening of the physical properties of muscle tissue of the long-arm octopus. However, as reported in a previous study on jumbo squid [50], the effect of heating raw squid on muscle tissue softening provided evidence to support the softening mechanism for the physical properties of long-arm octopuses.

The preference evaluation of the LOJP by elderly consumers showed that among appearance, taste, flavor, and texture items, preference for texture was the highest at 8.7 points. This can be attributed to the decrease in hardness (from 2153.6 × 1000 N/m^2^ to 34.4 × 1000 N/m^2^) under heating conditions (121 °C, 20 min). Torres-Arreola et al. [50] and Ando et al. [51] showed that heat treatment of the arrow squid (*Loligo bleekert*) mantle and jumbo squid (*Dosidicus gigas*) muscles softened the physical properties and increased the flavor components, improving palatability, which is consistent with our research results.

## 5. Conclusions

Herein, the seasoning sauce for long-arm octopus *Jangjorim* was produced by optimizing the mixing ratio of purified water, starch syrup, and soy sauce through RSM to develop HMR food suitable for elderly consumers. Various nutritional, physicochemical, and sensory characteristics of the LOJP were analyzed and compared with those of the long-arm octopus, which is the main raw material of the LOJP. The LOJP, with softened physical properties and enhanced umami because of the addition of soy sauce, a seasoning sauce, received a high score in the preference evaluation of elderly consumers and was suitable for the grade (physical property level 2) that can be consumed through the gums by elderly consumers. The results of this study may provide a basis for future studies on the development of HMR foods, not only for elderly consumers in Korea, but also for elderly consumers worldwide who have difficulty eating with their teeth.

## Figures and Tables

**Table 1:** Experimental range and values of independent variables in the CCRD for optimization of mixing ratio concentration of long-arm octopus *Jangjorim*.

Symbol	Range Level				
	−1.414	−1	0	+1	+1.414
*X*_1_	0.01	0.30	1.00	1.70	1.99
*X*_2_	0.09	0.50	1.50	2.50	2.91

*X*_1_, A/(B + C); *X*_2_, B/C; A, water (50%, *w*/*w*); B, starch syrup (30%, *w*/*w*); C, soy sauce (20%, *w*/*w*).

**Table 2:** Central Composite Rotatable Design of independent variables and response of dependent variables for preparation of long-arm octopus *Jangjorim*.

Run Number		Blending Ratio (%, *w*/*w*)			Independent Variables				Dependent Variables		
					Uncoded Values		Coded Values				
		A	B	C	*X*_1_	*X*_2_	*X*_1_	*X*_2_	*Y*_1_	*Y*_2_	*Y*_3_
Fractional factorial design (4 points)	1	17.8	10.7	71.5	−1	−1	0.22	0.15	49.3	8.59	3.2
	2	52.7	6.2	41.1	+1	−1	1.12	0.15	27.6	10.75	3.5
	3	17.8	37.8	44.4	−1	+1	0.22	0.85	34.9	7.11	2.7
	4	52.8	21.7	25.5	+1	+1	1.12	0.85	22.7	10.34	2.9
Axial portion (4 points)	5	2.9	32.4	64.7	−1.414	0	0.03	0.50	44.0	6.86	2.9
	6	56.6	14.5	28.9	+1.414	0	1.30	0.50	25.1	11.28	3.3
	7	40.0	0.3	59.7	0	−1.414	0.67	0.01	37.3	9.08	3.1
	8	40.0	29.9	30.1	0	+1.414	0.67	0.99	26.7	9.79	2.6
Center points (3 points)	9	40.0	20.0	40.0	0	0	0.67	0.50	27.1	10.61	4.5
	10	40.0	20.0	40.0	0	0	0.67	0.50	27.8	10.29	4.5
	11	40.0	20.0	40.0	0	0	0.67	0.50	26.8	11.10	4.2

*X*_1_, A/(B + C); *X*_2_, B/C; A, water; B, starch syrup; C, soy sauce; *Y*_1_: hardness (×1000 N/m^2^), *Y*_2_: salinity (g/100 g), *Y*_3_: sensory taste (points).

**Table 3:** Ingredients and content for the preparation of long-arm octopus *Jangjorim*.

Ingredients		Content (g)
Long-arm octopus arm		2000
Seasoning soy sauce	Water	154.0
	Starch syrup	308.0
	Soy sauce	256.9
	Cooking alcohol	154.0
	Starch water	359.1
Coconut oil		10.0
*Cheongyang* hot pepper		100.0
Konjac		100.0
Total		3442.0

**Table 4:** Estimated coefficients of the fitted quadratic polynomial equations for dependent variables based on the results of t-statistic.

Parameters	*Y*_1_		*Y*_2_		*Y*_3_	
	Coefficient	*p*-Value	Coefficient	*p*-Value	Coefficient	*p*-Value
Constant	37.5	0.000	2.5333	0.000	4.600	0.000
*X*_1_	−6.5	0.000	−0.4725	0.000	0.660	0.005
*X*_2_	6.7	0.000	−0.6755	0.000	0.945	0.001
*X*_1_ *X*_1_	4.6	0.003	0.1646	0.031	−1.013	0.002
*X*_2_ *X*_2_	−2.1	0.058	0.3896	0.001	−1.063	0.001
*X*_1_ *X*_2_	−3.0	0.034	−0.0500	0.481	−0.100	0.630

*X*_1_, A/(B + C); *X*_2_, B/C; A, water; B, starch syrup; C, soy sauce; *Y*_1_: hardness (×1000 N/m^2^), *Y*_2_: salinity (g/100 g), *Y*_3_: sensory taste (points).

**Table 5:** ANOVA test for response of dependent variables.

Dependent Variables	*p*-Value					R^2^
	Model	Linear	Quadratic	Cross-Product	Lack of Fit	
*Y*_1_	0.000	0.000	0.003	0.034	0.152	0.955
*Y*_2_	0.000	0.000	0.002	0.481	0.114	0.973
*Y*_3_	0.001	0.001	0.002	0.630	0.154	0.927

*Y*_1_: hardness, *Y*_2_: salinity, *Y*_3_: sensory taste.

**Table 6:** Optimal conditions predicted for preparation of long-arm octopus *Jangjorim* obtained by MINITAB program.

Dependent Variables	Values	*X*_1_		*X*_2_	
*Y*_1_	Target	37.0	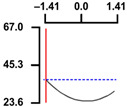	37.0	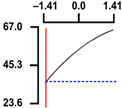
	Coded	−1.41		−1.37	
	Actual	0.01		0.13	
*Y*_2_	Target	2.5	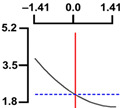	2.5	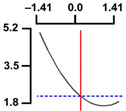
	Coded	0.00		0.05	
	Actual	1.00		1.55	
*Y*_3_	Target	Max.	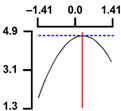	Max.	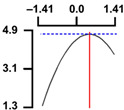
	Coded	0.30		0.44	
	Actual	1.21		1.94	
Multiple response optimization	Coded	0.07		0.00	
	Actual	1.05		1.50	
		A, 51.2% (*w*/*w*); B, 29.3% (*w*/*w*); C, 19.5% (*w*/*w*)			

*X*_1_, A/(B + C); *X*_2_, B/C; A, water; B, starch syrup; C, soy sauce; *Y*_1_: hardness (×1000 N/m^2^), *Y*_2_: salinity (g/100 g), *Y*_3_: sensory taste (points).

**Table 7:** Verification of predicted values for mixing ratio in preparation of the long-arm octopus *Jangjorim*.

Dependent Variables	Predicted Values	Experimental Values
*Y*_1_ (hardness, ×1000 N/m^2^)	37.0 ^a^	36.7 ± 1.3 ^a^
*Y*_2_ (salinity, g/100 g)	2.5 ^a^	2.5 ± 0.1 ^a^
*Y*_3_ (sensory taste, points)	4.7 ^a^	4.6 ± 0.1 ^a^

The letter a to the right of the data indicates no significant difference at the *p* > 0.05 level.

**Table 8:** Proximate composition, energy, and salinity of raw long-arm octopus arm and long-arm octopus *Jangjorim* prototype (LOJP).

Component		Raw	LOJP
Proximate composition (g/100 g)	Moisture	89.6 ± 0.5 ^b^	80.0 ± 0.2 ^a^
	Crude protein	8.7 ± 0.0 ^a^	9.7 ± 0.0 ^b^
	Crude lipid	0.6 ± 0.0 ^a^	4.2 ± 0.0 ^b^
	Ash	0.6 ± 0.1 ^a^	1.7 ± 0.1 ^b^
	Carbohydrate	0.5	4.2
Energy (kcal)		44.5	96.6
Salinity (g/100 g)		0.3 ± 0.0 ^a^	2.5 ± 0.0 ^b^

Carbohydrate (%) = 1—0 − (crude lipid + crude protein + ash + moisture). Energy (kcal) = (crude lipid × 9.02) + (crude protein × 4.27) + (carbohydrate × 3.87). Different letters (a, b) by data in the same row indicate significant differences at *p* < 0.05.

**Table 9:** Total amino acid contents (mg/100 g) and ratio (%) of raw long-arm octopus arm and long-arm octopus *Jangjorim* prototype (LOJP).

EAA	Raw	LOJP	NEAA	Raw	LOJP
Threonine	379.3 (4.6)	440.1 (5.0)	Aspartic acid	849.7 (10.2)	966.1 (10.9)
Valine	384.4 (4.6)	468.9 (5.3)	Serine	373.5 (4.5)	408.4 (4.6)
Methionine	168.8 (2.0)	159.2 (1.8)	Glutamic acid	1298.9 (15.7)	1417.4 (16.1)
Isoleucine	434.4 (5.2)	440.7 (5.0)	Proline	444.8 (5.4)	463.7 (5.3)
Leucine	631.9 (7.6)	639.3 (7.2)	Glycine	546.9 (6.6)	864.5 (9.8)
Phenylalanine	493.7 (6.0)	369.6 (4.2)	Alanine	465.9 (5.6)	543.8 (6.2)
Histidine	189.8 (2.3)	222.9 (2.5)	Cysteine	50.3 (0.6)	52.7 (0.6)
Lysine	748.1 (9.0)	707.3 (8.0)	Tyrosine	182.5 (2.2)	69.7 (0.8)
Arginine	654.0 (7.9)	590.4 (6.7)	Sub-total	4212.5 (50.8)	4786.3 (54.3)
Tryptophan	ND	ND	Total	8299.9 (100.0)	8824.7 (100.0)
Sub-total	4084.4 (49.2)	4038.4 (45.7)			

NEAA, non-essential amino acid; EAA, essential amino acid; ND, not detected. The value in parenthesis means the percentage of each AA compared to total AA content.

**Table 10:** Mineral contents (mg/100 g) of raw long-arm octopus arm and long-arm octopus *Jangjorim* prototype (LOJP).

Component	Raw	LOJP
Calcium (Ca)	19.1 ± 0.1 ^b^	23.7 ± 0.2 ^a^
Potassium (K)	144.2 ± 0.2 ^b^	375.7 ± 1.6 ^a^
Iron (Fe)	3.8 ± 0.0 ^a^	4.6 ± 0.0 ^b^
Zinc (Zn)	1.1 ± 0.0 ^b^	2.4 ± 0.0 ^a^

Different letters (a, b) by data in the same row indicate significant differences at *p* < 0.05.

**Table 11:** Fatty acid (FA) contents (mg/100 g) and ratio (%) of raw long-arm octopus arm and long-arm octopus *Jangjorim* prototype (LOJP).

FA	Raw	LOJP	FA	Raw	LOJP
4:0	ND	4.0 (0.2)	22:1n-9	0.8 (0.2)	13.5 (0.5)
11:0	32.5 (9.6)	ND	24:1n-9	ND	2.6 (0.1)
12:0	ND	1.8 (0.1)	ƩMUFA	36.5 (10.7)	170.9 (6.6)
14:0	3.6 (1.1)	15.3 (0.6)	18:2n-6	1.9 (0.6)	156.9 (6.1)
15:0	0.3 (0.2)	8.2 (0.3)	18:3n-3	0.2 (0.1)	7.5 (0.3)
16:0	56.5 (16.7)	344.4 (13.4)	20:2	1.2 (0.4)	33.3 (1.3)
17:0	4.8 (1.4)	100.7 (3.9)	20:3n-6	0.2 (0.1)	ND
18:0	23.2 (6.9)	383.5 (15.0)	20:3n-3	1.0 (0.3)	16.9 (0.7)
20:0	0.2 (0.1)	3.9 (0.2)	20:4n-6	ND	6.1 (0.2)
21:0	0.2 (0.1)	2.6 (0.1)	22:2	0.7 (0.2)	3.7 (0.1)
22:0	16.0 (4.7)	413.1 (16.1)	20:5n-3	79.8 (23.6)	263.2 (10.3)
ƩSFA	137.3 (40.8)	1277.5 (49.9)	22:6n-3	78.7 (23.3)	626.4 (24.5)
14:1	0.1 (0.0)	ND	ƩPUFA	163.7 (48.6)	1114.0 (43.5)
16:1	4.7 (1.4)	15.1 (0.6)	Ʃω-6	2.1 (0.7)	163.0 (6.3)
17:1	ND	2.4 (0.1)	Ʃω-3	159.7 (47.3)	914.0 (35.8)
18:1n-9	12.2 (3.6)	88.0 (3.4)	Total FA	337.5 (100.1)	2562.4 (100.0)
20:1n-9	18.7 (5.5)	49.3 (1.9)	Total Lipid	0.6 (56.1)	4.2 (61.0)

ND, not detected; 20:5n-3, DHA; 22:6n-3, EPA; SFA, saturated fatty acid; MUFA, monounsaturated fatty acid; PUFA, polyunsaturated fatty acid. The value in parentheses is the ratio of each FA to the total FA.

**Table 12:** The pH, VBN, TBC, BGC, and heavy metal of raw long-arm octopus arm and long-arm octopus *Jangjorim* prototype (LOJP).

Component		Raw	LOJP
Freshness indicators	pH	6.60 ± 0.0 ^a^	6.87 ± 0.0 ^b^
	VBN (mg/100 g)	11.1 ± 0.0 ^a^	15.4 ± 0.4 ^b^
	TBC (CFU/g)	1.3 × 10^3 b^	ND
	BGC (CFU/g)	-	ND
Heavy metals (μg/kg)	Lead (Pb)	0.01 ± 0.00 ^b^	0.002 ± 0.0001 ^a^
	Cadmium (Cd)	0.04 ± 0.00 ^b^	0.003 ± 0.0002 ^a^

ND, not detected. Different letters (a, b) by data in the same row indicate significant differences at *p* < 0.05.

**Table 13:** Acceptability evaluation of raw long-arm octopus arm and long-arm octopus *Jangjorim* prototype (LOJP).

Contents	A (Raw)	B (Boiled)	C (Sterilized)
Appearance	6.4 ± 0.7 ^a^	7.3 ± 0.7 ^ab^	8.4 ± 0.7 ^b^
Taste	6.3 ± 0.8 ^a^	7.1 ± 0.8 ^ab^	8.1 ± 0.7 ^b^
Flavor	5.6 ± 0.6 ^a^	7.6 ± 0.9 ^b^	8.0 ± 0.7 ^b^
Texture	4.2 ± 0.7 ^a^	7.3 ± 0.8 ^b^	8.7 ± 0.5 ^c^
Overall acceptance	5.6 ± 0.3 ^a^	7.3 ± 0.4 ^b^	8.3 ± 0.4 ^c^

Number of panels, n = 28 (14 females and 14 males); the average age, 70 (65–75); mean ± S.D. Different letters (a–c) by data in the same row indicate significant differences at *p* < 0.05.

## Data Availability

The data used to support the findings of this study can be made available by the corresponding author upon request.

## Supplementary Materials

The following supporting information can be downloaded at: https://www.mdpi.com/article/10.3390/foods12244375/s1, Figure S1: Operation method of the texture property meter in level 1 of texture. Figure S2: Operation method of the texture property meter in level 2 and level 3 of texture.
