# Supplementary material for: Development and Quality Characteristics of Jangjorim Prepared Using Long-Arm Octopus (Octopus minor) as an Elderly-Friendly Food
Source: Foods. 2023 Dec 5;12(24):4375. doi: 10.3390/foods12244375 (PMC10742802; doi:10.3390/foods12244375)
Supplement: Supplementary file 1 [file foods-12-04375-s001.zip › foods-2728446-supplementary.pdf]

foods

Supplementary material

Development and Quality Characteristics of *Jangjorim* Prepared Using Long-Arm Octopus (*Octopus minor*) as an Elderly-Friendly Food

Sang-In Kang[1], Jin-Soo Kim[2], Sun-Young Park[2], Si-Hyeong Park[2], Ji-Hoon Park[2], Mi-Soon Jang[3], Jae-Young Oh[3] and Jae-Suk Choi[2,*]

1 Seafood Research Center, Industry-Academic Cooperation Foundation, Silla University, 606, Advanced Seafood Processing Complex, Wontang-ro, Amanam-dong, Seo-gu, Busan, 49277, Republic of Korea; ftrnd5@silla.ac.kr (S.-I.K.)
2 Department of Seafood Science and Technology, Institute of Marine Industry, Gyeongsang National University, 2-9, Tongyeonghaean-ro, Tonyeong-si, 53064, Republic of Korea; jinsukim@gnu.ac.kr (J.-S.K.); tjsdud3591@gnu.ac.kr (S.-Y.P.); sipark@gnu.ac.kr (S.-H.P.); rnd-jh@gnu.ac.kr (J.-H.P.)
3 Food Safety and Processing Research Division, National Institute of Fisheries Science, 216, Gijanghaean-ro, Gijang-eup, Busan 46083, Republic of Korea; suni1@korea.kr (M.-S.J.); ojy0724@korea.kr (J.-Y.O.)
* Correspondence: jsc1008@gnu.ac.kr; Tel.: +82-55-772-9142

Supplementary materials

Experiment method 1

Grade 1 (tooth intake; can be ingested using teeth) senior-friendly food is a regular product; therefore, in the case of a sample for measuring its hardness, a sample with a size of 20 mm or more and wider than the area of the probe was used without pretreatment, such as cutting, if possible. The "Experiment method 1" of hardness measurement conditions using a rheometer are as follows. A compression speed of 100 mm/min and a sample temperature of 20±2 ℃ were set using a circular probe having a diameter of 5 mm and an acrylic bottom plate for measuring physical properties (thickness of 10 mm, center diameter of 10 mm hole). The depth was measured by completely penetrating the test product. The measured value was expressed as the average of three measurements after measuring the sample five times, excluding the maximum and minimum values (Figure S1).

Experiment method 2

The hardness measurement conditions using the rheometer for the 2nd step (taken with gums) and 3rd steps (taken with the tongue without teeth) of the elderly friendly food products were as follows. A container with a diameter of 40 mm and height of 2 mm was filled with the sample to a height of 15 mm. The hardness of the filled sample was measured using a circular probe with a diameter of 20 mm under the conditions of a compression speed of 600 mm/sec, a clearance of 5 mm, and a sample temperature of 20 ± 2 °C. The measured value is expressed as the average of three measurements after measuring the sample five times, excluding the maximum and minimum values (Figure S2).

Experiment method 3

The sample preparation method for measurement was the same as that in Experimental Method 1. The strengths of the samples were measured at a compression rate of 600 mm/min using a circular probe with a diameter of 3 mm. At this time, the temperature of the sample was 20 ± 2 °C. The clearance (the interval between the probe and sample) was set to 30% of the thickness of the sample. Each sample was measured five times under these conditions, and the average value of the three measurements, excluding the maximum and minimum values, is shown.

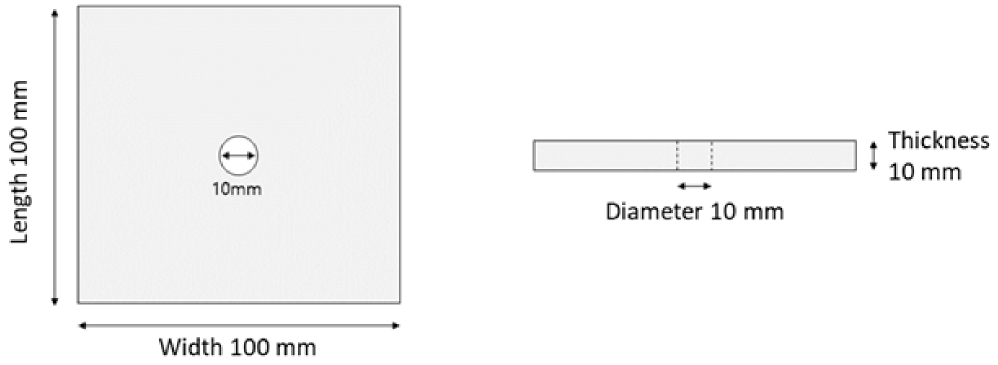

Plate for measurement of texture properties

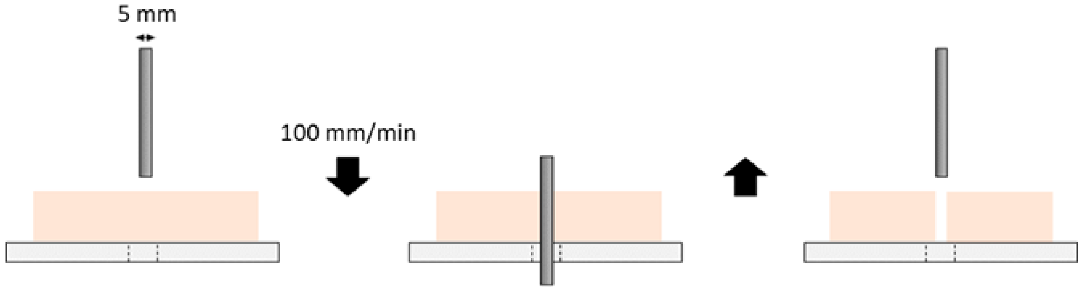

Operation in accordance with the "experiment method 3"

Figure S1. Operation method of the texture property meter in level 1 of texture.

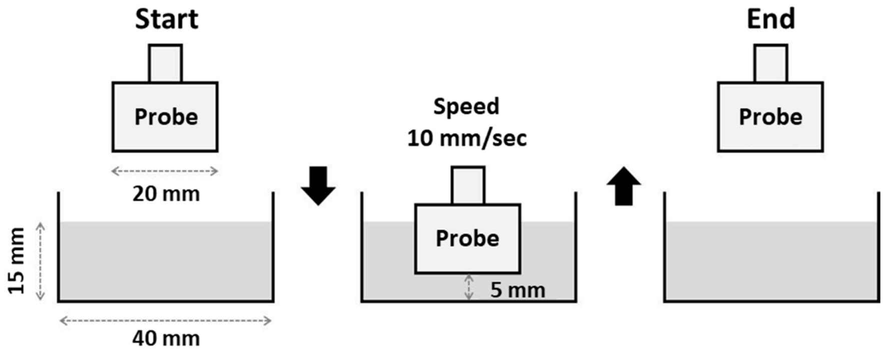

Figure S2. Operation method of the texture property meter in level 2 and level 3 of texture.
